# Inpatient non-communicable disease patterns and influencing factors at two tertiary hospitals in New Zealand and China: a mixed-methods study protocol

**DOI:** 10.1080/16549716.2026.2717007

**Published:** 2026-08-17

**Authors:** Yanhua Liu, Mengxue Zeng, Xingwen Luo, Oratanasataporn Soonthree

**Affiliations:** a Division of Health, University of Waikato, Hamilton, New Zealand; b West China School of Nursing, Sichuan University, Chengdu, China; c Department of Thoracic Surgery, West China Hospital, Sichuan University, Chengdu, China; d Department of Emergency Medicine, The Third People’s Hospital of Yibin, Yibin, China

**Keywords:** Chronic disease, hospitalisation, comparative health systems, mixed-methods, social ecological model

## Abstract

Non-communicable diseases (NCDs), including cardiovascular diseases, cancers, diabetes and chronic respiratory diseases, represent a growing inpatient burden worldwide. Yet hospital-based cross-national mixed-methods comparisons remain scarce, and the factors shaping inpatient NCD care are poorly understood. This protocol describes an explanatory sequential mixed-methods study examining inpatient NCD characteristics, disease patterns and hospitalisation indicators in tertiary hospitals in New Zealand and China, and exploring multilevel influences on NCD care. Guided by the Social Ecological Model, the study will be conducted at Waikato Hospital and the Third People’s Hospital of Yibin. In the quantitative phase, de-identified discharge records of adult inpatients aged ≥ 16 years with a primary diagnosis of one of the major NCDs will be extracted for 2023–2025. Analyses will be stratified by NCD category, and multivariable regression models with site as a fixed effect will examine variations in hospitalisation outcomes. In the qualitative phase, semi-structured interviews will be conducted with approximately 30–40 healthcare professionals across both settings. Interview data will be analysed thematically and integrated with quantitative findings using joint displays. To our knowledge, this is the first mixed-methods comparison of inpatient NCD care between tertiary hospitals in China and New Zealand. Findings are expected to support cross-cultural learning and inform evidence-based strategies to improve inpatient NCD management across diverse healthcare systems. This protocol is reported in accordance with the GRAMMS, STROBE and COREQ guidelines.

## Paper Context

- **Main findings**: This protocol sets out a disease-stratified, institution-level mixed-methods comparison of hospitalised non-communicable disease care at two tertiary hospitals.
- **Added knowledge**: This study protocol provides a hospital-based comparative framework integrating routinely collected data with qualitative insights from healthcare professionals to explain differences in chronic disease management between two distinct national healthcare systems.
- **Global health impact for policy and action**: Findings may offer actionable evidence to inform improvements in care coordination and discharge planning, supporting targeted hospital interventions that improve patient outcomes across diverse international settings.

## Background

Non-communicable diseases (NCDs), primarily including cardiovascular diseases, cancers, chronic respiratory diseases and diabetes, remain the leading contributors to global morbidity and mortality [1]. According to the World Health Organisation (WHO), NCDs account for 43 million deaths worldwide, representing approximately 75% of all deaths [2]. Their growing burden is driven by population ageing, urbanisation and changes in behavioural risk factors and has placed sustained pressure on health systems across countries at different stages of socioeconomic development [3,4]. Although NCD prevention and long-term management are commonly discussed in community and primary care settings, NCDs also generate a substantial inpatient burden through acute exacerbations, complications and complex multimorbidity, particularly among adults and older people [5–7].

Hospitals therefore represent a critical setting in which the contemporary burden of NCDs is most visible. Hospital discharge data provide a valuable source for characterising the inpatient NCD population, including patient demographic and clinical profiles, disease patterns and comorbidity, as well as hospitalisation indicators such as length of stay, in-hospital mortality and readmission. Previous research has used hospital administrative discharge data to investigate chronic disease patterns and associated hospitalisation outcomes in large patient cohorts [8]. Importantly, inpatient NCD care is shaped not only by clinical factors but also by how services are organised and delivered, such as care coordination, discharge planning and continuity of care [4,9]. Examining inpatient NCD patterns and related hospital care processes can thus provide insights into both health service performance and clinical practice.

Despite a large body of NCD research, important evidence gaps remain in relation to inpatient characteristics, health outcomes and determinants. Many existing studies focus on single diseases, single institutions or single-country settings and often rely on quantitative results alone. While such analyses can describe patterns and associations, they offer limited capacity to explain why patterns differ across settings or how multilevel influences shape inpatient NCD care [10,11]. Hospital-related cross-national comparisons and mixed-methods studies that integrate routinely collected inpatient data with qualitative perspectives remain scarce, and further work is needed to support explanatory understanding across contexts.

China and New Zealand offer an instructive contrast for examining inpatient NCD care. Both face substantial NCD-related morbidity and increasing demand for hospital services. Yet, they differ in key contextual features such as healthcare financing, service delivery structures and the distribution of health inequities [12,13]. In China, rapid population ageing and persistent urban–rural disparities contribute to complex NCD-related hospital needs [14,15]. While in New Zealand, the burden is compounded by enduring inequities in health outcomes, particularly among Māori and Pacific peoples [16,17]. Studying tertiary hospitals operating within these contrasting systems provides a meaningful basis for examining which inpatient NCD patterns are shared and which are shaped by local context.

To address this gap, our study adopts an explanatory sequential mixed-methods design, underpinned by the Social Ecological Model (SEM) as the guiding theoretical framework [18,19]. We aim to examine both shared patterns and differences in inpatient NCD profiles and outcomes between Waikato Hospital and the Third People’s Hospital of Yibin, analysed separately within each major NCD category, and to explore how multilevel factors contribute to these findings. We do not aim to draw national-level inferences; comparisons are confined to these two institutions and interpreted within their respective system contexts. The study seeks to generate evidence on inpatient NCD patterns and the health-service factors that shape them, supporting service improvement and cross-system learning.

## Objectives and research questions

### Objectives

This study aims to: (1) describe and compare hospitalised NCD patient characteristics, disease patterns and hospitalisation indicators at Waikato Hospital (New Zealand) and the Third People’s Hospital of Yibin (China); (2) explore healthcare professionals’ perspectives on multilevel factors influencing inpatient NCD care in each setting; (3) integrate quantitative and qualitative findings, guided by the SEM, to explain both shared patterns and differences in inpatient NCD care and to inform health service improvement.

### Overall research question

What are the similarities and differences in hospitalised NCD patient characteristics, disease patterns, hospitalisation indicators and social-ecological determinants in Waikato and Yibin, and how can these shared patterns and context-specific variations be explained?

### Specific research question

#### Quantitative phase

- What are the demographic and clinical characteristics of hospitalised NCD patients at each hospital?
- How do NCD profiles and hospitalisation indicators (e.g. length of stay, in-hospital mortality and readmission rates) compare between the two hospitals?
- Which patient-level and hospital-level factors are associated with variations in hospital outcomes among inpatient NCD populations?

#### Qualitative phase

- Which individual and interpersonal factors (e.g. health literacy, family support and lifestyle) do healthcare professionals identify as influencing inpatient NCD care in Waikato and Yibin?
- How do institutional and system-level factors (e.g. clinical pathways, workforce composition and discharge planning) shape inpatient NCD management in each setting?
- How do community and policy factors (e.g. healthcare accessibility, insurance arrangements and public health initiatives) influence the hospital experience of patients with NCDs?

The qualitative questions are designed to provide explanatory insight into the patterns and variations identified in the quantitative phase.

## Methods

### Research design

**Figure 1.**
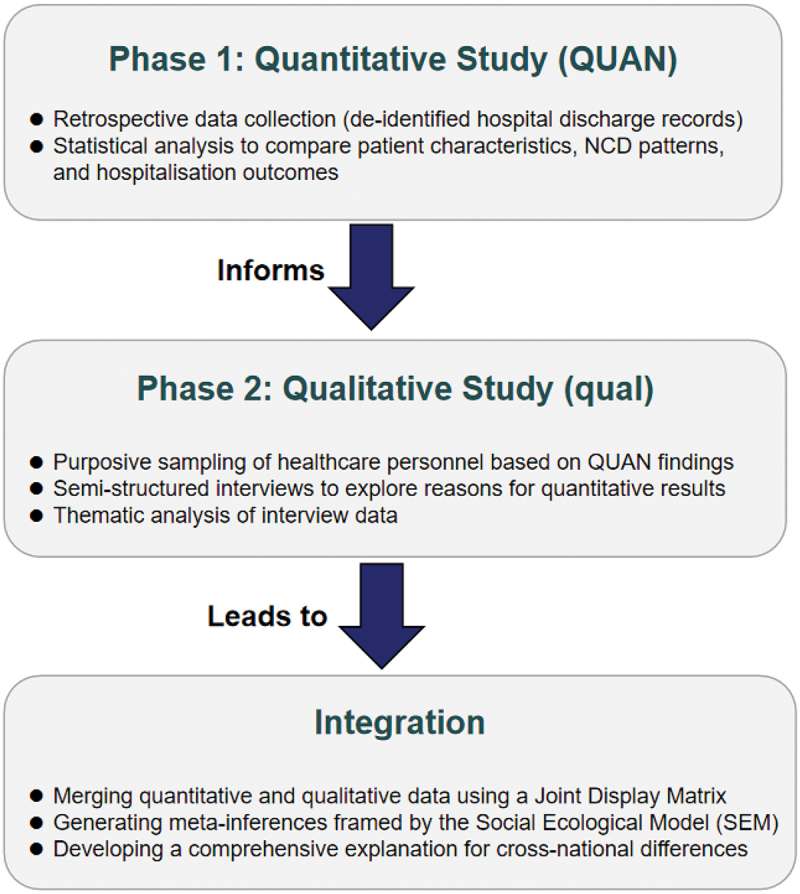
Study design flowchart.

This study adopts an explanatory sequential mixed-methods design (QUAN → qual), guided by the pragmatism paradigm. As illustrated in Figure 1, the process begins with the collection and analysis of quantitative data to identify overall features in inpatient NCD characteristics and hospitalisation indicators. Subsequently, qualitative data will be collected and analysed to explain, clarify or expand upon the quantitative findings [20]. A pragmatist approach is well suited to this design because it supports the integration of quantitative and qualitative methods to address complex, practice-oriented health research questions [21].

### Theoretical framework

**Figure 2.**
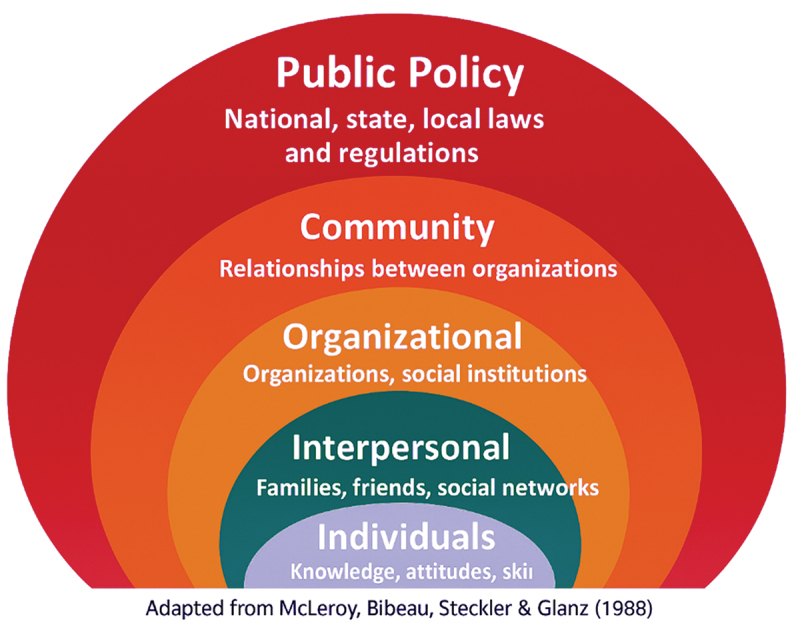
The social ecological model framework.

The Social Ecological Model (SEM) provides the interpretive framework for this study (Figure 2) [19,22]. Rather than driving statistical model specification, the SEM is used to organise the interview guide and to structure the integration of quantitative and qualitative findings. In the quantitative phase, variables derived from hospital discharge data are described at the level at which they are recorded; we do not claim that administrative variables fully capture social-ecological constructs. For example, insurance type is treated as an individual-level financing characteristic rather than as a direct measure of health policy. In the qualitative phase, the semi-structured interview guide is organised around the five SEM levels to support systematic exploration of multilevel influences on inpatient NCD care. During integration, the SEM serves as the interpretive lens for relating quantitative findings (‘what’) to qualitative themes (‘why’), supporting a multilevel explanatory account of inpatient NCD patterns and outcomes. We do not test statistical cross-level interactions or fit a multilevel SEM, because the two-site design does not support such modelling.

## Research site

This study will be conducted at Waikato Hospital in New Zealand and the Third People’s Hospital of Yibin in China (Table 1). Waikato Hospital is a major public tertiary hospital serving the Waikato region of New Zealand [23]. It provides care to a diverse population, including Māori and Pacific peoples who experience persistent health inequities, making it a relevant setting for examining inpatient NCD care in New Zealand [24]. The Third People’s Hospital of Yibin is a tertiary general hospital located in Sichuan Province, China, serving a large urban and rural catchment population [25]. As a regional referral hospital in southwest China, it provides an appropriate setting for examining inpatient NCD patterns within the Chinese healthcare system.

**Table 1.** Study sites: Waikato Hospital (New Zealand) and the Third People’s Hospital of Yibin (China), with key hospital indicators.

Indicator	Waikato Hospital (NZ)	Yibin Third People’s Hospital (China)
Location	Hamilton, Waikato region	Yibin, Sichuan Province
Beds	759	750
Hospital level	Major tertiary referral centre	Municipal tertiary general hospital
Key specialties	Trauma; Cardiology; Oncology; Tertiary obstetrics	Neurology (stroke centre); Oncology; Cardiology; Intensive care unit
Population served	Waikato region — mixed urban–rural catchment	Yibin prefecture and surrounding counties
Annual admissions	~55,000	~31,000
Notes on coding	ICD-10-AM	ICD-10 (local variant)

Abbreviation: ICD-10-AM: International Statistical Classification of Diseases and Related Health Problems, Tenth Revision, Australian Modification.

## Phase 1: quantitative research (QUAN)

### Population and sampling

This phase will include de-identified discharge records for adult inpatients (aged ≥ 16 years) with a primary diagnosis of one of the four major NCDs, including cardiovascular diseases (ICD-10 codes I00-I99), cancers (C00-C97), diabetes (E10-E14) and chronic respiratory diseases (J40-J47), discharged from Waikato Hospital and the Third People’s Hospital of Yibin between 1 January 2023 and 31 December 2025. Records will be excluded if patients were aged ≤ 15 years, admissions were limited to non-admitted emergency department observation visits, diagnostic codes did not meet the predefined NCD definitions, or essential variables were missing.

### Data collection and variables

Quantitative data will be collected using a census approach, including all eligible inpatient discharge records during the study period. Because this captures the entire eligible inpatient population at each site, no a priori sample size calculation is required. Based on hospital admission records, the eligible inpatient population is estimated at approximately 41,000 records at Waikato Hospital and 32,000 at the Third People’s Hospital of Yibin. For the multivariable analyses, adequacy is therefore assessed in terms of the number of outcome events relative to the number of covariates. Even under conservative assumptions for the lowest-frequency outcome (in-hospital mortality), the expected number of events comfortably exceeds the commonly cited threshold of 10–20 events per candidate variable for the planned models, supporting stable estimation across the pre-specified covariates. Where a disease stratum yields a small number of events, results will be interpreted with appropriate caution or the model simplified accordingly.

A standardised data extraction template will be used to obtain de-identified discharge data from the electronic medical record systems of both hospitals. Key variables will include patient demographics, clinical characteristics and hospitalisation indicators, as summarised in Table 2. To ensure comparability between settings, an ICD-10 code mapping framework has been developed to harmonise potential diagnostic coding differences between the two countries.

**Table 2.** Summary of key quantitative variables for data extraction.

Variable Category	Key Variables
Patient Demographics	Age, Sex, Ethnicity/Nationality, Urban/Rural Residence, Insurance Type^1^, Education Level^2^
Clinical Characteristics	Primary NCD Diagnosis (ICD-10), Secondary Diagnoses, Comorbidities (Charlson Comorbidity Index), Admission Type (Emergency/Elective)
Hospitalisation Indicators	Length of Stay (LOS), In-Hospital Mortality, 30-Day Readmission Rate, Discharge Destination

Abbreviation: NCD: Non-communicable Disease; ICD-10: International Statistical Classification of Diseases and Related Health Problems, Tenth Revision; ^1^Insurance type is recorded as an individual-level financing characteristic and is not used as a measure of health policy. ^2^Education level is inconsistently recorded in administrative data, particularly at the New Zealand site; it will be analysed only where reliably available, with missingness reported.

### Data analysis

Quantitative data will be analysed with Stata 18. Descriptive statistics will summarise patient demographic and clinical characteristics at each hospital. All comparative analyses will be stratified by NCD category (cardiovascular disease, diabetes, cancer, chronic respiratory disease); no pooled cross-disease outcome will be computed, so that differences in case-mix between sites are not misattributed to site or system differences. Within each disease group, categorical variables will be compared using chi-square tests and continuous variables using independent-samples t-tests or Mann–Whitney U tests according to distribution.

Multivariable regression will examine associations between patient-level factors and hospitalisation outcomes, with site included as a fixed effect; logistic regression will be used for binary outcomes and Cox proportional hazards regression for time-to-event outcomes. Models will be pre-specified rather than selected by stepwise procedures, adjusting for a fixed set of confounders (age, sex, comorbidity burden and admission type). Because some patients contribute more than one admission, cluster-robust (sandwich) standard errors at the patient level will be used to account for within-patient correlation. Formal multilevel modelling with a hospital-level random effect is not appropriate given only two sites, as the higher-level variance cannot be reliably estimated; instead, site is modelled as a fixed effect and pre-specified interaction terms (for example, site by age group) and sensitivity analyses will be used to examine effect modification and robustness. Missing data will be reported by variable and site; variables judged missing at random will be handled by multiple imputation in the comparative models, with complete-case sensitivity analyses.

## Phase 2: qualitative research (QUAL)

### Participants and sampling

A purposive maximum variation sampling strategy will be employed to recruit approximately 15–20 healthcare professionals per site (a total of approximately 30–40 participants across the two hospitals). As an indicative target, we will seek a majority of nurses (approximately half of each site sample) alongside medical specialists, general practitioners and allied health staff, with final numbers determined by maximum-variation sampling and data saturation rather than fixed quotas. Nurses are prioritised given their central role in coordinating inpatient NCD care, monitoring patient deterioration and facilitating discharge planning across both settings. Participants may be based in hospital or community settings where their roles relate to the delivery, coordination or follow-up of inpatient NCD care. Individuals who are currently interns or trainees, or whose roles are exclusively administrative, will be excluded. This approach is intended to ensure diversity in professional roles, work settings and levels of experience. Recruitment will continue until sufficient data saturation is achieved, defined as no new SEM-relevant themes arising across three consecutive interviews, assessed jointly by two coders.

### Data collection

Qualitative data will be collected through face-to-face, one-on-one semi-structured interviews conducted in a quiet office or teaching space, with interviews lasting approximately 45–60 minutes. The interview guide will be structured around the five levels of the SEM and will be informed by key findings from the quantitative phase to facilitate in-depth exploration of factors underlying observed patterns (Supplementary File: Interview Guide). With written informed consent, interviews will be audio-recorded, transcribed verbatim and de-identified prior to analysis.

### Data analysis

NVivo 14 will be used to support data management and thematic analysis. Interview recordings will be transcribed verbatim. To preserve meaning, coding will be conducted on the original-language transcripts (Chinese or English) by bilingual members of the research team; forward and back translation will be used for the cross-team discussion and reporting of themes and illustrative quotations, rather than as the basis for coding.

Analysis will combine a deductive framework based on the five SEM levels, applied first to organise the data, with inductive open coding to capture themes not anticipated by the framework; the two approaches will be reconciled during theme development. Coding will be conducted independently by multiple researchers, with discrepancies resolved through discussion and consensus. Qualitative analysis will focus on generating explanatory insights into quantitative findings; for example, where quantitative analyses identify differences in multimorbidity, qualitative data will be used to explore potential cultural, economic and organisational explanations.

### Data integration and triangulation

Quantitative and qualitative data will be integrated sequentially through connection, merging and interpretation phases [26,27].

#### Connection

Findings from the quantitative phase will inform qualitative sampling and the refinement of interview questions, with particular attention to statistically significant differences, unexpected results or outliers [20,28].

#### Merging

A joint display will be used to integrate quantitative results with corresponding qualitative themes [29]. A worked template is provided as Supplementary Table S1. The display juxtaposes each quantitative finding, the related qualitative evidence, an integration judgement and the resulting meta-inference. Integration judgements are defined as convergence (quantitative and qualitative findings agree), expansion (qualitative findings explain or extend the quantitative result) and discordance (findings conflict). Where discordance arises, we will return to both datasets, seek disconfirming evidence and resolve the interpretation through team discussion before finalising the meta-inference [27].

#### Interpretation

Meta-inferences will be developed to generate integrated insights that extend beyond single data sources. These interpretations will be structured across the five levels of the SEM to elucidate how multilevel factors interact to shape inpatient NCD care patterns and outcomes [18]. Qualitative findings will be explicitly mapped to relevant SEM levels and integrated with quantitative results to support a coherent, theory-informed explanatory model and targeted recommendations for NCD management.

## Reporting standards

This study protocol will be reported in accordance with relevant EQUATOR guidelines for mixed-methods research, including the Good Reporting of a Mixed Methods Study (GRAMMS) guideline [30]. Quantitative and qualitative components will be conducted and reported following established standards for observational and qualitative research, respectively. Integration procedures will be transparently described to ensure clarity and reproducibility.

## Ethics considerations

This study will be conducted in accordance with the principles of the Declaration of Helsinki. Ethical approval for this study has been obtained from the University of Waikato Human Research Ethics Committee (Ethics Approval No: HREC(Health)2025#83) and the hospital ethics committees of the Third People’s Hospital of Yibin (Ethics Approval No:2025042). The quantitative phase will use de-identified routinely collected hospital discharge data. For the qualitative phase, written informed consent will be obtained from all participants prior to interviews. Confidentiality and anonymity will be maintained throughout the study.

## Trial and protocol registration

This study does not involve a clinical trial and therefore does not require registration in a clinical trial registry. The study protocol has been registered and is publicly available on the Open Science Framework (OSF) (Registration DOI:10.17605/OSF.IO/7ECZY).

## Discussion

This explanatory sequential mixed-methods protocol outlines a hospital-based approach to examining inpatient NCD patterns, hospitalisation indicators and multilevel influencing factors across two contrasting healthcare systems. By integrating routinely collected hospital discharge data with qualitative insights from healthcare professionals and applying the SEM as an interpretive framework, the study is intended to move beyond descriptive comparisons and generate explanatory understanding of both shared patterns and context-specific variations in inpatient NCD care.

The study is likely to identify a combination of common features and setting-specific differences in the characteristics and hospitalisation profiles of patients admitted with NCDs. At the New Zealand site, national and regional evidence suggests that ethnicity-related inequities, particularly among Māori and Pacific peoples, may be reflected in inpatient NCD patterns [31]. At the Yibin site, inpatient profiles may more strongly reflect rapid population ageing and behavioural risk factors documented in the Chinese context [32]. Differences in hospitalisation indicators, such as length of stay and in-hospital mortality, may also be observed between settings, even after accounting for variation in age and comorbidity. Differences in service organisation, such as care coordination, discharge planning and the integration of chronic disease management, are also likely to emerge between the two settings. China has expanded integrated chronic NCD management clinics as one response to its rising NCD burden [33], and contrasting such service models with the New Zealand setting may help explain observed differences in hospitalisation patterns. These factors will be explored in the qualitative phase to identify transferable opportunities for service improvement.

The qualitative phase is expected to provide explanatory insight into these patterns by elucidating multilevel contextual influences on inpatient NCD care. Factors operating at individual and interpersonal levels, such as patient resources and support networks, may interact with institutional arrangements, healthcare financing mechanisms and service accessibility to shape care processes and hospital experiences [34–36]. Through the integration of quantitative and qualitative findings, the SEM-guided analysis will support interpretation of how cultural, social and policy conditions interact to influence inpatient NCD patterns across the two settings.

The findings of this study have the potential to inform clinical practice, service planning and health system improvement. By comparing inpatient NCD care across two healthcare environments, the study may identify areas of shared challenge as well as specific opportunities for improving care delivery. These insights may inform service-level interventions, strengthen care coordination and contribute to efforts to address health inequities and promote cross-system learning in the management of NCDs. Findings will be framed as institution-level evidence and explicitly contextualised against published national and regional data rather than presented as nationally representative.

## Strengths and limitations

This study offers several strengths. It adopts a hospital-based cross-national comparative design to examine inpatient NCD patterns across two contrasting healthcare systems. The explanatory sequential mixed-methods approach enables quantitative findings to be complemented by qualitative insights, enhancing explanatory depth. In addition, the SEM provides a coherent interpretive framework for interpreting and integrating findings across multiple levels of influence.

Several limitations should also be acknowledged. First, the two participating hospitals cannot represent national healthcare status in China or New Zealand. Findings are therefore confined to the study sites and should not be interpreted as nationally representative. National and regional data will be used only to provide contextual background. Differences in annual admission volume further support a site-specific interpretation. Second, measurement bias may arise from differences in diagnostic coding practices. This risk will be reduced through standardised ICD-10 mapping and sensitivity analyses. Because coding systems and case-mix differ between sites, the Charlson Comorbidity Index will be reported descriptively within each site rather than treated as a directly comparable cross-site measure. Similarly, discharge destination will be analysed within site only. Third, social desirability bias may influence qualitative interviews. This will be mitigated by emphasising confidentiality and using neutral, open-ended questions. The multilingual composition of the research team may also affect interpretation; this risk will be addressed through bilingual coding and team discussion. Fourth, residual confounding from unmeasured factors may remain despite adjustment for known covariates. Finally, patient and caregiver perspectives are beyond the scope of this provider-focused study and warrant further investigation in future research.

## Conclusion

This protocol addresses an important gap in hospital-based research by proposing a mixed-methods comparison of inpatient NCD patterns and multilevel influences between tertiary hospitals in China and New Zealand. Findings are expected to enhance understanding of factors influencing inpatient NCD care and may inform future strategies for improving care coordination, patient management and discharge planning across different healthcare settings.

## Supplementary Material

Supplementary File Interview Guide.docx

GRAMMS Checklist GHA.docx

Supplementary Table S1.docx

## Data Availability

The protocol is registered and publicly available on the Open Science Framework (DOI: 10.17605/OSF.IO/7ECZY). This research is a methodological protocol and does not involve quantitative or qualitative datasets.
