# Supplementary material for: Inpatient non-communicable disease patterns and influencing factors at two tertiary hospitals in New Zealand and China: a mixed-methods study protocol
Source: Glob Health Action. 2026 Aug 17;19(1):2717007. doi: 10.1080/16549716.2026.2717007 (PMC13482924; doi:10.1080/16549716.2026.2717007)
Supplement: Supplementary file 1 [file ZGHA_A_2717007_SM5582.docx]

**Supplementary Table S1.** Illustrative joint display for integrating quantitative and qualitative findings, organised by Social Ecological Model level.

*Note: The entries below are illustrative only and do not represent study findings. They show how each quantitative result will be juxtaposed with the related qualitative evidence, assigned an integration judgement, and developed into a meta-inference. Integration judgements are defined as convergence (quantitative and qualitative findings agree), expansion (qualitative findings explain or extend the quantitative result) and discordance (findings conflict). Where discordance arises, the team will return to both datasets, seek disconfirming evidence and resolve the interpretation through discussion before finalising the meta-inference.*

| **SEM level** | **Illustrative quantitative finding** | **Illustrative qualitative theme / evidence** | **Integration judgement** | **Meta-inference** |
| --- | --- | --- | --- | --- |
| **Individual** | Within the diabetes stratum, admitted patients at one site show a higher mean comorbidity burden and later-stage presentation. | Professionals describe low health literacy and delayed care-seeking among many patients before admission. | **Expansion** | Individual-level knowledge and care-seeking behaviour may partly account for differences in admission complexity, beyond clinical case-mix. |
| **Interpersonal** | Within the cardiovascular stratum, 30-day readmission rates are broadly similar across the two sites. | At both sites, professionals emphasise strong family caregiving involvement in post-discharge support. | **Convergence** | Comparable family-level support across settings may help explain the similarity in early readmission. |
| **Organisational (institutional)** | Within the respiratory stratum, length of stay is longer at one site after adjustment for age and comorbidity. | Professionals at that site cite bed pressure and gaps in discharge coordination. | **Expansion** | Institutional discharge processes appear to shape length of stay independently of clinical severity. |
| **Community** | Readmission is higher among patients recorded as rural-residing. | Professionals describe limited community follow-up and transport barriers for these patients. | **Convergence** | Access to community-level resources after discharge may influence readmission risk. |
| **Social policy** | The two sites differ in the mix of elective versus emergency admissions. | Professionals relate this to differences in insurance and reimbursement arrangements and in gatekeeping to hospital care. | **Expansion** | Financing and access policies appear to shape admission pathways; an interpretation tested against the descriptive data. |
| **Across levels (discordant example)** | Within the cancer stratum, in-hospital mortality does not differ significantly between sites. | Professionals at one site nonetheless perceive poorer outcomes for these patients. | **Discordance** | Perception and recorded outcome diverge. We will return to both datasets, seek disconfirming evidence and examine case-mix and unmeasured factors before finalising the inference. |

Abbreviation: SEM, Social Ecological Model.
