# Supplementary material for: Inpatient non-communicable disease patterns and influencing factors at two tertiary hospitals in New Zealand and China: a mixed-methods study protocol
Source: Glob Health Action. 2026 Aug 17;19(1):2717007. doi: 10.1080/16549716.2026.2717007 (PMC13482924; doi:10.1080/16549716.2026.2717007)
Supplement: Supplementary file 2 [file ZGHA_A_2717007_SM5583.docx]

**GRAMMS Checklist**

**Good Reporting of A Mixed Methods Study (GRAMMS)**

Manuscript: Inpatient Non-Communicable Disease Patterns and Influencing Factors at Two Tertiary Hospitals in New Zealand and China: A Mixed-Methods Study Protocol

| **No.** | **GRAMMS Item** | **Where addressed in the manuscript** |
| --- | --- | --- |
| 1 | Describe the justification for using a mixed methods approach to the research question | Background (final paragraph) and Methods – Research design: an explanatory sequential mixed-methods design is justified on the grounds that quantitative discharge data alone cannot explain why inpatient NCD patterns differ between settings, so qualitative data from healthcare professionals are required to provide explanation (pp. 2, 5). |
| 2 | Describe the design in terms of the purpose, priority and sequence of methods | Methods – Research design and Fig. 1: explanatory sequential design (QUAN → qual); priority given to the quantitative phase, followed sequentially by the qualitative phase, which is used to explain the quantitative findings (p. 5). |
| 3 | Describe each method in terms of sampling, data collection and analysis | Phase 1 – Quantitative (population and census sampling, data extraction variables in Table 2, stratified analysis with site as a fixed effect, pp. 8–10) and Phase 2 – Qualitative (purposive maximum-variation sampling of 15–20 professionals per site, semi-structured interviews, NVivo 14 deductive–inductive thematic analysis, pp. 11–12). |
| 4 | Describe where the integration has occurred, how it has occurred and who has participated in it | Data integration and triangulation: integration occurs across connection, merging and interpretation phases; a joint display (Supplementary Table S1) links quantitative findings to qualitative themes with convergence/expansion/discordance judgements; the research team jointly develops meta-inferences, with discordance resolved through team discussion (p. 12). |
| 5 | Describe any limitation of one method associated with the presence of the other method | Strengths and Limitations: limitations arising from mixing methods are acknowledged, including potential contamination between phases (qualitative prompts shaped by quantitative findings) addressed through open, non-leading prompts, and the multilingual research team’s potential effect on interpretation addressed through bilingual coding and team discussion (p. 15). |
| 6 | Describe any insights gained from mixing or combining methods | Discussion: the value of integrating routinely collected discharge data with provider perspectives is described, explaining how qualitative insight will help interpret why hospitalisation patterns differ and identify transferable, context-specific opportunities for service improvement that neither method alone could reveal (p. 14). |

*Page numbers refer to the clean (revised) manuscript and may shift slightly after typesetting.*

*Reference: O’Cathain A, Murphy E, Nicholl J. The quality of mixed methods studies in health services research. J Health Serv Res Policy. 2008;13(2):92–98.*
