# Supplementary material for: Inpatient non-communicable disease patterns and influencing factors at two tertiary hospitals in New Zealand and China: a mixed-methods study protocol
Source: Glob Health Action. 2026 Aug 17;19(1):2717007. doi: 10.1080/16549716.2026.2717007 (PMC13482924; doi:10.1080/16549716.2026.2717007)
Supplement: Supplementary file 3 [file ZGHA_A_2717007_SM5584.docx]

Supplementary File: Interview Guide

**Interview Outline for Health Professionals (ENG)**.

**Interviewee demographics**

Participant ID..................................;

Years of experience: .................................;

Main responsibilities/ target audience: ..................................;

Primary professional role: ▢Registered Nurse	▢General Practitioner	▢Specialist Physician	▢Dietitian, ▢Other

Speciality/Department:  ▢Cardiology	▢Respiratory	▢Endocrinology	▢Oncology  ▢General Practice	▢Community Health

**Introduction**

Thank you for agreeing to speak with me. This interview is part of my PhD research comparing NCD inpatient care in New Zealand and China. I'm interested in your professional experiences and perspectives. The interview will last about 45-60 minutes. Please remember this is confidential, and you can skip any question or stop at any time. Do you have any questions before we begin?

Professional Background (5 min)

- “Can you describe your current role, department, and years of experience?”
- “What led you to specialise in [cardiology/endocrinology/oncology/respiratory medicine]?”

| Level | Purpose | Questions | | Responses |
| --- | --- | --- | --- | --- |
| Individual  (5 min) | Explore how personal knowledge, skills, and attitudes shape care  challenges | 1. Please describe the key characteristics of typical inpatients with cardiovascular disease, diabetes, cancer, or chronic respiratory disease that you encounter in your daily work.   - Probe: age distribution, comorbidities, complications; recent change; | |  |
|  |  | 2. What do you think are the biggest challenges patients face in disease management (e.g., medication adherence, blood sugar/blood pressure monitoring, lifestyle changes, etc.)?   - Probe: Health literacy influence; Common misconception among patients? | |  |
|  |  | Further site-comparison probes to be added following quantitative analysis, per the connection phase. | |  |
| Interpersonal  (10 min) | Understand team dynamics and patient–family interactions | 3.“How does family involvement typically affect an NCD patient's journey, both before and after hospitalisation?”   - Probe: Are family burdens, caregiving conflicts, or costs (e.g., loss of income) common? Do they affect medication use/follow-up appointments? | |  |
|  |  | 4. “What role does social support (or lack thereof) play in patient outcomes? Can you give any examples?”   - Probe: family meetings, inclusive decision‑making. | |  |
|  |  | Further site-comparison probes to be added following quantitative analysis, per the connection phase. | |  |
| Organisational  (15 min) | Identify how hospital structures, processes, and resources facilitate or impede care | Care Pathways & Processes | 5.“Please describe your hospital/department's routine diagnosis, treatment, and discharge procedures for patients with NCDs (including multidisciplinary consultation, discharge education, and follow-up arrangements).”   - Probe: How do bed availability and emergency department volume influence hospitalisation decisions? Under what circumstances would a patient typically be admitted to a hospital rather than managed in the community? Are there any issues with information transfer or document gaps? |  |
|  |  |  | 6.“Which parts of these procedures work smoothly, and where are the bottlenecks?” |  |
|  |  | Resources & Infrastructure | 7.“What are the strengths of your hospital's approach to NCD care? What are the biggest challenges (e.g., staffing, resources, processes)?”   - Probe: ICU beds, rehab services, electronic records. |  |
|  |  | Discharge &Referral | 8.“In terms of discharge preparation and community integration, which measures have been effective, and which often fail? Could you provide specific examples of both successful and unsuccessful cases?”   - Probe: What pressures influence the timing of your decision to discharge patients? Is there a dedicated discharge coordinator or community liaison available? What is the general follow-up rate? |  |
|  |  |  | 9.“What measures has the hospital taken to improve the quality of NCD care? How effective do you think these measures are?” |  |
|  |  | Further site-comparison probes to be added following quantitative analysis, per the connection phase. | |  |
| Community  (5 min) | Explore external services and environmental factors affecting patients | 10. “After discharge, what community resources (e.g., family physicians, community health services, rehabilitation/pharmacy services) are available to support hospital readmissions and management of complications?” How accessible are these resources?   - Probe: How do transportation, geography, or economic factors influence patient retention in care? Are community health promotion programs common and effective? | |  |
|  |  | 11. “In your view, how do broader community factors (e.g., access to healthy food, safe places for exercise, local culture) influence the NCD burden you see in the hospital?” | |  |
|  |  | Further site-comparison probes to be added following quantitative analysis, per the connection phase. | |  |
| Social Policy  (10 min) | Examine the influence of health policies and funding models | 12. “In what ways do you think current medical insurance/reimbursement, drug supply, or national chronic disease management policies help or hinder effective patient management? (Please provide examples.)   - Probe: For example, how do drug accessibility, prescription restrictions, reimbursement rates, and prescription renewal processes impact clinical practice? | |  |
|  |  | 13. “Have recent policy changes affected patient admission patterns or treatment choices?” | |  |
|  |  | 14. “Are there challenges in the implementation of health policies at the organisational or clinical level? If so, what factors contribute to these challenges?” | |  |
|  |  | 15.“What policy reforms would you advocate for to improve NCD inpatient care?” | |  |
|  |  | 16. “What cultural values or social norms do you believe influence patients’ healthcare-seeking behaviours, treatment adherence, or hospitalisation experiences?”   - Probe: Population’s perceptions of ageing, families’ sense of responsibility in caregiving, and public expectations regarding the state’s health responsibilities, among other factors. | |  |
|  |  | Further site-comparison probes to be added following quantitative analysis, per the connection phase. | |  |

Cultural & Equity Considerations (5 min)

- Purpose: Assess how cultural and equity issues shape care delivery.
- “How does your department address the needs of Māori/Pacific patients (NZ) or ethnic minorities (China)?”
- Probe: culturally tailored services, interpreter use, liaison roles.
- “Can you share an example where cultural sensitivity improved—or lack of it hindered—patient care?”

Reflections & Suggestions (5 min)

- “Based on your experience, what are the top three changes that could enhance non-communicable disease inpatient care?”
- “How might nursing practice, interdisciplinary teamwork, or hospital policy evolve to better support these patients?”
- “What actionable advice or best practices can you share at the hospital, community, or policy level?”

Closing (2 min)

- Thank them for their time and insights.
- Remind them of withdrawal rights (up to 2 weeks).
- Ask if they have any final questions or comments.

***Note:*** *The above are merely the common basic questions proposed based on the SEM theoretical framework. More questions regarding the differences between the two study settings will be added to the interview outline after quantitative data analysis.*
